# Disparities in Pancreatic Cancer Treatment and Outcomes

**DOI:** 10.1089/heq.2019.0057

**Published:** 2019-10-29

**Authors:** Marcus Noel, Kevin Fiscella

**Affiliations:** Department of Medicine Hematology and Oncology Division, University of Rochester Medical Center, Wilmot Cancer Institute, Rochester, New York.

**Keywords:** pancreatic neoplasms, health care disparities, epidemiology

## Abstract

**Purpose:** Pancreatic cancer remains a major health concern; in the next 2 years, it will become the second leading cause of cancer deaths in the United States. Health disparities in the treatment of pancreatic cancer exist across many disciplines, including race and ethnicity, socioeconomic status (SES), and insurance. This narrative review discusses what is known about these disparities, with the goal of highlighting targets for equity promoting interventions.

**Methods:** We performed a narrative review of health disparities in pancreatic cancer spanning greater than ten areas, including epidemiology, treatment, and outcome, using the PubMed NIH database from 2000 to 2019 in the Unites States.

**Results:** African Americans (AAs) tend to present at diagnosis with later stage disease. AAs and Hispanics have lower rates of surgical resection, are more likely to be treated at low volume hospitals, and often experience higher rates of morbidity and mortality compared to white patients, although control for confounders is often limited. Insurance and SES also factor into the delivery of treatment for pancreatic cancer.

**Conclusion:** Disparities by race and SES exist in the diagnosis and treatment of pancreatic cancer that are largely driven by race and SES. Improved understanding of underlying causes could inform interventions.

## Introduction

Pancreatic cancer is among the deadliest forms of cancer. It is the seventh most common malignancy, but currently represents the third leading cause of cancer deaths in the United States.^1^ It is estimated that in 2019, 45,750 patients will die from pancreatic cancer (3490 more than breast cancer), and by 2020, it will become the second leading cause of cancer death.^1^ Risk factors for pancreatic cancer include smoking, diabetes, obesity, chronic pancreatitis, and family history.^2^ Over 80% of patients present with metastatic disease.^2^ Despite advances in chemotherapy, the average survival remains <1 year.^3^ For those patients who are able to undergo resection, the 5-year survival rate increases to only 25–30%.^2^

Similar to other common malignancies, pancreatic cancer is associated with disparities by socioeconomic status (SES), ethnic minority status, and insurance.^4,5^ In contrast to other types of cancer (breast, colon) where screening can detect early-stage disease, no screening modality exists for pancreatic cancer. Thus, disparities in outcomes for pancreatic cancer do not result from lack of screening.^6^

We conducted a narrative review to examine health disparities related to pancreatic cancer. We adopted the Centers for Disease Control and Prevention definition for health disparities: “Health disparities are differences in health outcomes between groups that reflect social inequalities.”^7^ This definition includes social factors such as SES, geography, insurance, and so on. Thus, we reviewed the literature to determine if there were racial and/or ethnic differences in care and outcomes for pancreatic cancer patients and if social factors contributed to differences by race and/or ethnicity.

There have been relatively few reviews of disparities in pancreatic cancer and none this comprehensive. This review will serve as a general outline of current disparities in pancreatic cancer and highlight areas that can be focused on to close the gap in care over the next several years.

## Methods

We conducted a narrative review of the literature on pancreatic treatment and outcomes, including contribution by social factors. We conducted multiple searches using the PubMed (NIH) database (2000–2019). The search was limited to human studies published in English. Keyword combinations of the medical subject headings (MeSH) included “pancreatic cancer,” “pancreatic neoplasm,” “disparities,” “ethnicity,” and “insurance.” We included disparities related to race and ethnicity, as well as SES and insurance. We began by briefly reviewing differences in epidemiology, then reviewing disparities in rates of surgical resection, surgical morbidity, chemotherapy and radiation for advanced disease, referral patterns, and declined treatment. We conducted secondary searches by reviewing the references of primary articles and references to primary articles to identify additional articles for inclusion and critical review. We excluded studies conducted outside of the United States and those published before 2000.

## Results

We present our results organized into three main categories, epidemiology, treatment, and outcome. Within each major category, we will review disparities as they pertain to race and ethnicity, SES, and insurance. We begin with reviews of disparities in the epidemiology of pancreatic cancer and stages at diagnosis to better understand the context, including the role of potential biological factors.

### Disparities in pancreatic cancer epidemiology

#### Incidence

Earlier studies showed that African Americans (AAs) have a 50–90% higher incidence of pancreatic cancer compared to other racial groups.^8^ Some studies fail to explain the higher incidence among AAs, and others note differences by race/ethnicity and varying levels of poverty.^9,10^ Another recent study conducted in Georgia found that AAs had a significantly higher age-adjusted incidence (14.6 per 100,000) compared to whites (10.8 per 100,000). A meta-analysis found 40% higher rates among AAs compared to whites.^11^ The association of incidence with poverty may not be linear. In one study, the incidence per 100,000 for the high, medium, and low poverty groups was 9.2, 9.9, and 9.5, respectively.^12^ A recent study may shed additional light on racial differences, discovering that somatostatin subtype receptor (SSTR5) is recognized as a regulator of pancreatic tissue and that genotype differences in SSTR5 may exist by race.^13–15^ Studies have not adequately assessed the contribution of behavioral or genetic risk factors to racial differences in incidence.

#### Stage at diagnosis

Stage at the time of diagnosis is the most important variable for pancreatic cancer survival. According to the latest Surveillance, Epidemiology, and End Results (SEER) data, the stage at diagnosis slightly favors white patients compared to black, although this difference is not statistically significant. From 2004 to 2010, 37% and 52% of white patients presented with loco regional and distant disease versus 34% and 57% of AA patients.^16^ Differences in tumor biology may contribute to a more advanced stage at diagnosis for AAs.^17^ Clinicopathologic analysis of AA patients compared to white patients is notable for a higher presence of K-mutations at codon 12 and less frequent FAS expression, a finding described in other malignancies.^18^ Further study is needed to ascertain the role, if any, of these biological factors.

### Disparities in treatment

#### Referrals to cancer specialists

Using the SEER rates of consultation with a cancer specialist, evaluation was determined by race.^19^ AAs were significantly less likely, compared to whites, to see a medical oncologist (AA 52.6% vs. white 60.2%, *p*<0.001), radiation oncologist (AA 25.6% vs. white 32.5%, *p*<0.05), or a surgeon (AA 72.1% vs. white 78%, *p*≤0.01). The reasons for lower referral rates are not clear.

#### Surgical resection

For the 15–20% of patients who present with early-stage disease, surgery offers the only option for cure.^2^ Several studies have evaluated the varying rates of resection for eligible patients based on a number of variables, including ethnicity and insurance status.

Using the California Cancer Registry (CCR), when pancreatic cancer was identified, rates of disease were 38% and 37% for white and black patients, respectively.^20^ However, a higher proportion of whites underwent resection compared to AAs (42% vs. 36%, *p*=0.002). For those with resectable disease, white race, younger age, and non-Medicare/Medicaid insurance predicted undergoing resection. AAs were 34% less likely to undergo resection compared to whites (odds ratio [OR]=0.66, 95% confidence interval [CI]: 0.54–0.080).

A second study using three registries: CCR, the Cancer Surveillance Program of Orange County, and the San Diego Imperial Organization for Cancer Control discovered that within subjects with early-stage disease, AAs were the most likely to not receive surgery regardless of the staging method used (80% AA, 67.8% non-Hispanic white, 62% Hispanic).^21^ Despite similar rates of insurance coverage, only 25% of AAs underwent resection compared to 30.7% of non-Hispanic whites and 39.5% of Hispanics.

A third group found similar results using the SEER database from 1992 to 2002.^8^ Blacks and whites were recommended for surgery at similar rates, 34% versus 34.5%, respectively. However, blacks underwent significantly fewer resections (10.6% vs. 12.7%, *p*<0.001).

A fourth study used the National Cancer Database of the American College of Surgeons to determine socioeconomic factors in receipt of pancreatic surgery for resectable disease. Surgery was offered more frequently to white patients compared to black patients (27.5% vs. 22.9%, *p*<0.001).^22^

As surgical technique continues to evolve, minimally invasive surgery (MIS) has become an option. Gabriel and colleagues found that 13.5% of 442,679 patients underwent MIS. Analysis revealed that patients of Hispanic origin were less likely to undergo MIS of the body/tail (Hispanic vs. non-Hispanic, OR=0.24, 95% CI: 0.07–0.79, *p*=0.019).^23^

A group from Harbor-UCLA Medical Center used the SEER database to determine if median family income correlated with surgical resection.^24^ Seventy-one (33%) patients were resected in the low-income group in comparison to 679 (39.9%) in the middle income and 1827 (45.8%) in the high-income group. In addition, univariate analysis revealed a statistically significant difference in resection between patients in the low- and middle-income groups compared to high income (*p*=0.0001).

Finally, a study using data from Florida's cancer data system found that patients in the lowest SES category compared to the highest were less likely to have pancreatic cancer surgery (16.5% vs. 19.8%, *p*<0.001).^25^ It is important to note that most of these studies did not adjust for the presence and type of health insurance, much less individual level income. Insurance is associated with receiving resection.^22^

In 2006, Massachusetts instituted the health care reform act that provided insurance to nearly all residents in the state. Before this act, publicly insured and self-pay patients had significantly lower rates of pancreatic resection compared to privately insured patients. Using the Agency for Healthcare Research and Quality's State Inpatient Databases, Loehrer et al. compared patients who were admitted with pancreatic cancer in Massachusetts to those admitted to three other control states.^26^ The 2006 insurance expansion was associated with a 67% increased rate of pancreatectomy for public insurance/self-pay patients in Massachusetts relative to control states (incidence rate ratio=1.67, 95% CI: 1.01–2.76, *p*=0.043).

Shavers et al., using SEER, found that uninsured patients had a significantly lower adjusted OR of receiving surgery (OR=0.07, 95% CI: 0.01–0.49).^27^ A second study from the University of Massachusetts also found that counties with higher rates of uninsured patients, 65% versus 60.9%, had lower rates of surgery performed (among patients recommended for surgery that were statistically significant, *p*≤0.0001).^28^ These findings point to affordability, as indicated by insurance, as one possible social contributor to racial disparities in surgery. (Refer to Tables 1 and 2 for a summary of the previously mentioned studies).

**Table 1.** Disparities in Treatment for Pancreatic Cancer

Author	Year of publication	Years of study	Database	Number of patients	Ethnic disparity	SES	Insurance	Methods	Key findings
Disparities in rates of surgical resection									
Abraham^20^	2013	1994–2008	CCR	20,312	AA vs. white	N/A	Yes	Retrospective cohort analysis using multivariate logistic regression	Black and underinsured patients receive treatment that deviates from guidelines
Chang^21^	2005	1988–1998	CCR	16,679	AA vs. other race	N/A	N/A	Retrospective population based analysis	AAs had higher incidence and underwent less surgical treatment
Murphy^8^	2009	1992–2002	SEER	27,828	AA vs. white	N/A	Yes	Retrospective univariate analysis	Crude survival did not differ among races; multivariate analysis demonstrated a survival disadvantage for blacks
Shapiro^4^	2016	2004–2011	SEER	17,350	AA vs. white	N/A	Yes	Retrospective, cohort	Disparities are associated with SES
Cheung^25^	2010	1998–2002	Florida Cancer Registry	16,104	N/A	Yes	N/A	Retrospective, univariate, and multivariate analysis	Low SES less likely to receive standard treatment
Shavers^27^	2009	1998	SEER	2404	AA vs. Hispanic vs. white	N/A	Yes	Retrospective multivariate analysis	Differences in tumor characteristics did not explain all ethnic disparities
Moaven^22^	2019	1998–2012	NCDB	280,935	AA vs. white	Yes	Yes	Retrospective bivariate analysis	Race and insurance were the most important factors in receipt of surgery
Disparities in surgical resection based on hospital/surgeon volume									
Al-Refaie^30^	2012	2003–2008	National Inpatient Sample	59,181	Non-white vs. white			Retrospective bivariate analysis	Non-white race and increased comorbidities contribute to receipt of care at LVH
Epstein^31^	2010	2001–2004	NYC Hospital discharge Data	570	Non-white vs. white			Cross-section, regression analysis	Minority patients are more likely to use LVH
Eppsteiner^32^	2009	1998–2005	Nationwide Inpatient Sample	3581	AA vs. Hispanic vs. white	Yes		Retrospective, case–control	HV surgeons had lower adjusted mortality rates
Liu^67^	2006	2000–2004	California Discharge Database	719,608	Non-white vs. white		Yes	Retrospective database	Substantial disparities in characteristics of patients treated at HVH
Disparities in rates of surgical complications									
Lucas^47^	2006	1994–1999	Medicare Database	10,032	AA vs. white			Retrospective multiple logistic regression	Black patients have higher operative mortality
Sukumar^46^	2015	1999–2009	Nationwide Inpatient Sample	301,634	AA vs. white			Retrospective cross-sectional analysis	Racial disparities exist for black patients
Cheung^25^	2010	1998–2002	Florida Cancer Registry	16,104	N/A	Yes		Retrospective, univariate, and multivariate analysis	Low SES less likely to receive standard treatment
Disparities in rates on adjuvant treatment									
Shavers^27^	2009	1998	SEER	2404	AA vs. Hispanic vs. white			Retrospective multivariate analysis	Differences in tumor characteristics did not explain all ethnic disparities
Abraham^20^	2013	1994–2008	CCR	20,312	AA vs. white			Retrospective cohort analysis using multivariate logistic regression	Black and underinsured patients receive treatment that deviates from guidelines
Murphy^8^	2009	1992–2002	SEER	27,828	AA vs. white			Retrospective univariate analysis	Crude survival did not differ among races; multivariate analysis demonstrated a survival disadvantage for blacks
Disparities in rates of surgical refusal									
Eloubeidi^42^	2006	1996–2000	Alabama Statewide Cancer Registry	2230	AA vs. white			Retrospective Fisher exact test	Black patients were less likely to receive therapy, but were also more likely to refuse therapy
Murphy^8^	2009	1992–2002	SEER	27,828	AA vs. white			Retrospective univariate analysis	Crude survival did not differ among races; multivariate analysis demonstrated a survival disadvantage for blacks
Shah^44^	2013	1998–2009	SEER	35,944	AA vs. white			Retrospective, univariate, and multivariate analysis	Disparities in recommendations for pancreatic surgery
Disparities in survival									
Zell^51^	2007	1989–2003	CCR	24,735	AA vs. Hispanic vs. white	Yes		Retrospective case only analysis	Differences in treatment and SES likely account for poor survival in AA and Hispanic patients
Wray^52^	2012	1998–2010	Cancer Tumor Registry from 2 Hospitals	1039	AA vs. Hispanic vs. white			Retrospective univariate analysis	AAs found to have lowest survival

AA, African American; CCR, California Cancer Registry; HV, high volume; HVH, high surgical volume hospital; LVH, Low Volume Hospital; N/A, not applicable; NCDB, National Cancer Database; SEER, Surveillance, Epidemiology, and End Results; SES, socioeconomic status.

**Table 2.** Disparities in Surgical Resection/Chemotherapy

Author	Year of publication	Outcome	Results
Abraham^20^	2013	Rate of surgical resection	AA 36% vs. W 42% (*p*=0.002)
Chang^21^	2005	Rates of NOT undergoing resection	AA 80% vs. A 77.1%
			NWH 67% vs. H 62%
Murphy^8^	2009	Rate of surgical resection	AA 10.6% vs. W 12.7% (*p*<0.001)
Moaven^22^	2019	Rate of surgery offered	AA 22.9% vs. W 27.5% (*p*<0.001)
Shapiro^4^	2016	Rates of surgical resection	AA vs. W (OR=0.76, 95% CI: 0.65–0.88, *p*<0.001)
			I vs. NI (OR=1.63, 95% CI: 1.22–2.18, *p*=0.001)
Shavers^27^	2009	Receipt of chemotherapy	AA vs. NWH (OR=0.94, 95% CI: 0.92–0.96)
		Receipt of radiation	H vs. NWH (OR=0.50, 95% CI: 0.27–0.95)

A, Asian; CI, confidence interval; H, Hispanic; I, insured; NI, noninsured; NWH, non-white Hispanic; OR, odds ratio; P, *p*-value; W, white.

The cause of disparities in racial surgical resection is not clear, although insurance/affordability appears to be a likely contributor. Other factors such as quality of communication between providers and minority patients, patient preferences, and mistrust/fear have not been adequately assessed.

#### Hospital volume

Higher hospital surgical volume for pancreatic resection is associated with better outcomes. In a retrospective analysis of patients undergoing pancreatic resections, high surgical volume hospitals (HVHs) had statistically (*p*<0.00001) lower mortality (2.7%) compared with low volume hospitals (LVHs; 11.1%).^29^ In another study from North Carolina, mortality was significantly less at high volume centers (2.8%) compared to low (10.3%) from 2007 to 2009, OR=0.34.

Using the National Inpatient Sample (NIS), 59,841 patients underwent surgical resection.^30^ On bivariate analysis, Asian/Pacific Islander patients were most likely to have a pancreatectomy at a LVH of 57.3% (defined as less than 11 pancreatectomies a year) compared with 33.6% of white patients. Using multivariate analysis, non-white patients were more likely to receive resection at LVHs compared with whites (AA vs. white, OR=1.9, *p*<0.0001).

Epstein et al. also found disparities in receipt of care at LVHs versus HVHs.^31^ Among the 570 patients who had pancreatic surgery, 36% of white patients received surgery at a HVH (at least 47 procedures a year) with a high volume surgeon (at least 10 procedures a year), compared to only 19% of Asian and Hispanic patients and 10% of AA patients. Even after adjusting for demographics, including socioeconomic and insurance characteristics, the difference in rates of surgery at HVHs with high volume surgeons was statistically significant (*p*<0.05) between AA and white patients.

A retrospective analysis of surgeon volume found that compared to low volume surgeons' patients, those who underwent resection by high volume surgeons were more likely to be white race (81.1% vs. 74%) and treated in a teaching hospital (91.0% vs. 58.2%).^32^ Importantly, high volume surgeons had a lower adjusted mortality compared with low volume surgeons (2.4% vs. 6.4%, *p*<0.0001). These findings suggest the role of a potential social factor that contributes to disparities in outcomes, hospital, and surgical volume for pancreatic resection. The factors driving these differences, such as distance to a HVH or patient/family preference for a particular hospital, are unknown.

#### Adjuvant treatment

In 2004, a large randomized trial demonstrated a survival benefit with adjuvant chemotherapy, setting the standard for current treatment.^33,34^ Abraham et al. found that after adjusting for covariates of age, race, extent of disease, and nodal positivity, all variables continued to predict chemotherapy receipt.^20^ Interestingly, AA patients were 25% less likely to receive adjuvant chemotherapy (OR=0.75, 95% CI: 0.58–0.98). In addition, AA patients were 30% less likely to receive adjuvant chemoradiation (OR=0.71, 95% CI: 0.53–0.95).

Using the SEER database, 2404 patients with a diagnosis of pancreatic cancer were identified in 1998.^27^ In the adjusted logistic regression model, AAs were statistically less likely to receive chemotherapy (OR=0.61, 95% CI: 0.37–0.95) compared to non-Hispanic whites. Among those with advanced disease, AAs were 30% less likely to receive primary chemotherapy compared to whites (OR=0.69, 95% CI: 0.60–0.80) and 50% less likely to receive primary chemotherapy plus radiation (OR=0.53, 95% CI: 0.41–0.70).^20^ The role of the patient or clinician and system level factors in these disparities are not clear.

#### Advanced disease

For patients with unresectable disease, the current standard of care includes systemic chemotherapy; previous recommendations, however, included chemotherapy combined with radiation. Abraham et al. report that white patients received chemotherapy and chemoradiation more frequently than AA patients did (42% vs. 37%, *p*=0.001; 10% vs. 6%, *p*<0.0001).^20^ Khanal et al. found that patients with private insurance (61.5%) were more likely to receive systemic therapy compared with those without insurance (*p*<0.01).^35^ In a previously mentioned study using Florida's cancer data system, patients in the lowest SES category compared to the highest were also less likely to receive chemotherapy (30.7% vs. 36.4%, *p*<0.001) or radiation treatment (14.3% vs. 16.9%, *p*=0.003).^25^ In summary, there are racial differences in chemotherapy for advanced disease. The causes for these differences are not clear from these data.

#### Geographic location

One study evaluated patients with locally advanced pancreatic cancer, identified using a SEER database, and assessed the impact of area of residence. Cancer directed therapy was received by 44% of the cohort; univariate analysis revealed that 56% of patients in the top socioeconomic stratum (based on SES for the area of residence) received treatment, compared to 35% in the lowest stratum.^36^

Yet another study found that using univariate analysis, the likelihood of having the OR for recommendation of no surgery was lowest in the Northeast (0.8), West (1.6), Southeast (1.3), and Midwest (ref) (*p*<0.05 for all).^37^ Regional differences have also been verified in other retrospective studies.^38^ Whether these regional differences primarily reflect source of care, clinician recommendation, patient preferences, social class, or affordability is not apparent from the data.

#### Declined treatment

Treatment options for pancreatic cancer are relatively straightforward. Patients with early-stage disease are offered resection and those with advanced disease are offered chemotherapy or radiation. Patients with poor performance status may appropriately decline treatment, but for those in otherwise good health, treatment tends to improve survival.

Patient willingness to undergo surgery has been hypothesized to contribute to disparities in surgery for cancer of the colon, esophagus, and lung.^39–41^ In a study of 2254 patients diagnosed with pancreatic cancer, authors found that across all tumor stages, AAs were slightly more likely to refuse therapy (5.6% vs. 2.9%, *p*=0.02 for chemotherapy and 9.0% vs. 3.3% for surgery, *p*=0.001).^42^

Murphy et al.^8^ and Tohme et al.^43^ found that AAs were also more likely to refuse surgery. However, it is not clear to what extent higher rates of refusals among AAs reflect suboptimal patient communication, patient perceptions of greater risk, greater mistrust, or unmeasured difference in morbidity/functional status. Shah et al. report that surgeons less often recommended resection for comparable stage among AAs.^44^ Further study is needed to explicate reasons for racial disparities in patient consent/refusal for surgery.

### Disparities in outcome

#### Surgical morbidity and mortality

For those patients who do undergo pancreatic resection, complications, delayed gastric emptying, and postoperative fistula are common. Morbidity is lower among experienced surgeons.^45^ Sukumar et al. found higher rates of surgical complications for blacks following pancreatic resection.^46^ After controlling for patient characteristics, including pancreatic resection comorbidity score, AA patients had higher mortality (OR=1.27, 95% CI: 1.01–1.61).^47^ Furthermore, after adjustment for hospital volume the significant mortality differences remained. However, a recent, well-controlled national study showed no differences in perioperative mortality by race/ethnicity.^48^ Similarly, a retrospective study using the CCR found no differences by race, ethnicity, or SES in survival following resection for stages I/II pancreatic cancer after controlling for age, sex, comorbidity, tumor stage and grade, type of surgery, chemotherapy, and surgical volume of the hospital.^49^

#### Survival

Historically, population based studies have reported poorer survival for AA patients with pancreatic cancer.^50^ Using the CCR, a retrospective study found that differences in treatment and SES likely account for the poor survival of AA and Hispanic patients.^51^ After adjusting for age, year of diagnosis, and gender, AAs (hazard ratio [HR]=1.14, 95% CI: 1.08–1.21) and Hispanics (HR=1.06, 95% CI: 1.01–1.11) had an increased rate of death compared to whites. However, once adjusting for treatment, the difference in survival was no longer statistically significant.

A retrospective survival analysis, which used the registry from two hospital systems, found that AAs had worse survival rates compared to Caucasian (HR=1.2, *p*=0.05) patients, even after adjusting for treatment.^52^ Another study completed at Massachusetts General Hospital found that AA (HR=1.1, *p*=0.01) and Hispanic (HR=1.2, *p*<0.01) patients had worse survival rates compared with white patients, even after adjustments for treatment.^53^ However, data that are more recent suggest nearly comparable survival statistics among white and black patients. For white patients with localized and distant disease, the 5-year survival rate was 25% and 7%, respectively, compared to 23% and 6% for black patients.^16^ A recent study that includes multiple controls reported similar survival rates.^22^ The finding of comparable outcomes following surgery (when controlling for surgical volume) may extend to some regions.^49^

## Discussion

In this narrative review, we evaluated cancer disparities related to pancreatic cancer. Our aim was to expand our review beyond that of AAs and include all races, Asian Americans, Hispanics, Native Americans, and others. Unfortunately, we found the literature lacking regarding non-black minority groups. Most of the studies compared AAs to whites. Previous studies have suggested that implicit bias contributes to treatment of other cancers.^54–56^ However, no studies were identified that directly addressed this question for pancreatic cancer.

Notably, findings from multiple studies show that AAs consistently have lower rates of surgical resection despite controlling for early-stage disease, yet comparable surgical outcomes when controlling volume. Potential explanations for different rates of resection based on race include differences in surgeon recommendations and a lack of patient understanding and trust.^57^ Cultural differences between patients and physicians can compromise trust or undermine confidence in care resulting in increased skepticism when consenting for surgery. Older studies have suggested that patient beliefs regarding the effects of air exposure on tumor spread may contribute to refusals.^58^

Additional factors contributing to racial disparities in surgical resection include frequent care of minority patients at LVHs and care by less specialized and/or experienced physicians. Furthermore, patients who live in areas that are more rural or live farther from a tertiary care center may not be able to travel for highly specialized medical care.^59^ Viable strategies that improve access to care at HVHs among minority patients might reduce disparities.

Poor access in health care may contribute to differences in stage at diagnosis. Patient navigation is a tool that has been used to reduce cancer disparities. In a study of 3777 cancer patients (breast, cervical, colorectal, and prostate) from 2007 to 2011, diagnostic delays were noted based on employment and housing status, and these disparities were eliminated through use of personal patient navigators.^60^ Pancreatic cancer is a rapidly progressive disease, thus patient navigation might facilitate a more rapid diagnostic work-up and possibly diagnose more patients at an earlier stage. There are some data suggesting that delayed diagnosis is associated with poor prognosis in pancreatic cancer.^61^ Research is needed to determine whether reducing the time between symptoms and diagnosis improves survival and reduces disparities in outcomes.

Additional specific interventions, targeting disparities in outcomes from pancreatic cancer, may make a difference once effective screening strategies for pancreatic cancer are identified. The Delaware Cancer Consortium, founded in 2002, was designed with three key elements: a colorectal cancer screening program, a cancer treatment program for the uninsured, and an emphasis on AA cancer disparity reduction.^62^ The results of this program were notable for an increase in all colorectal cancer screening in Delaware in patients >50 years of age from 57% in 2002 to 74% in 2009, and screening rates for AAs rose from 48% to equal the 74% rate among whites during the same period. Although screening modalities are currently lacking in pancreatic cancer, other principles implemented in this Consortium, including treatment programs for the uninsured, could possibly reduce disparities in pancreatic cancer as well (e.g., through more rapid evaluation of symptoms). Other solutions may include receipt of care in an integrated health system. A study from Kaiser Permanente found no disparities in care or outcomes for minorities compared with whites.^63^ Similarly, in an equal access system (U.S. Department of Defense), no racial disparities in treatment or survival for pancreatic cancer were observed.^64^

## Conclusion

Evidence suggests that AAs, and in some instances Hispanics, have lower rates of surgery, receive less aggressive stage specific treatment, and receive surgery at LVHs and/or by lower volume surgeons and that these differences might contribute to disparities in outcomes. Moreover, patients who are underinsured or uninsured also tended to receive less aggressive care. These findings suggest that socioeconomic, insurance, and geographic access factors might contribute to racial differences in treatment and outcomes. The role of access is further supported by studies from integrated and/or equal access systems of care showing no disparities in treatment or survival. Potentially, policies designed to improve early access to high quality treatment for minority patients could eliminate disparities in outcomes for pancreatic cancer.

Limitations to this review are reflected in our search, which was confined to articles published in English and with a focus on the United States. Other limitations include differences in study samples based on era, geography, and number and granularity of the measures included. Relevant studies may not have been included because of poor key wording or publication bias. We recognize that our findings cannot be generalized worldwide; however, underserved populations across the globe often face similar barriers to cancer care.

In summary, we observed racial disparities in treatment of pancreatic cancer similar to those for treatment of other cancers and for surgery for other conditions.^65,66^ The explanations appear multifactorial, but largely reflect inequalities in social factors. Possibly, these gaps will narrow with an increasing focus on improving equity in oncologic care and as more citizens gain health insurance through the Affordable Care Act. Regardless, patients and their clinicians should continue to advocate, to ensure that patients receive the highest level of care irrespective of race, income, or insurance type.

## Abbreviations Used

AAs: African Americans
CCR: California Cancer Registry
CI: confidence interval
HR: hazard ratio
HVHs: high surgical volume hospitals
LVHs: low volume hospitals
MIS: minimally invasive surgery
NCDB: National Cancer Database
NIS: National Inpatient Sample
NWH: non-white Hispanic
OR: odds ratio
SEER: Surveillance, Epidemiology, and End Results
SES: socioeconomic status
SSTR5: somatostatin subtype receptor
